# Provisioning of vitellogenic follicles continues after green turtles arrive at the nesting beach

**DOI:** 10.1093/conphys/coaf012

**Published:** 2025-02-24

**Authors:** Renato Saragoça Bruno, Alan B Bolten, Karen A Bjorndal

**Affiliations:** Department of Biology, Archie Carr Center for Sea Turtle Research, University of Florida, 882 Newell Dr., Gainesville, FL 32611, USA; Turtle Love, Barra de Parismina, Limón 70 301, 200m south of the river mouth, Lapa Verde Lodge, Costa Rica; Department of Biology, Archie Carr Center for Sea Turtle Research, University of Florida, 882 Newell Dr., Gainesville, FL 32611, USA; Department of Biology, Archie Carr Center for Sea Turtle Research, University of Florida, 882 Newell Dr., Gainesville, FL 32611, USA

**Keywords:** Follicular dynamics, green turtles, jaguar predation, multiple clutches, nutrient provisioning, reproductive investment, Tortuguero, vitellogenesis, yolk deposition

## Abstract

Understanding the energetic demands of reproduction on female sea turtles is crucial for devising effective conservation strategies aimed at supporting the reproductive health and resilience of populations at nesting habitats. We studied the ovaries of 69 green turtles (*Chelonia mydas*) preyed upon by jaguars (*Panthera onca*) during three nesting seasons at Tortuguero, Costa Rica, the main green turtle Atlantic nesting beach. Our findings revealed a bimodal distribution of vitellogenic follicles, with ‘dominant’ follicles destined for ovulation and ‘non-dominant’ follicles to be resorbed. Female green turtles lay, on average, six clutches with ~110 eggs each per nesting season, and a size hierarchy was also found within dominant follicles. During the nesting season, the diameter of small dominant follicles increased by 66% prior to ovulation. Analysis of yolk composition showed that small dominant follicles had higher percent water content than large dominant follicles, which indicates dry matter deposition rather than hydration is responsible for the pre-ovulatory increase in diameter of green turtle dominant follicles during the nesting season. Furthermore, percentages of lipid, nitrogen (N) and phosphorus (P) in the yolk dry matter were constant across green turtle vitellogenic follicles, which underscores that the increase in follicle size results from provisioning with yolk containing similar proportions of these nutrients. Atretic follicles had higher water and lower P percentages than dominant follicles, indicating an accelerated resorption of phosphorus over lipids and N, which could be due to the importance of this nutrient for eggshell production. Finally, >49% of the energy required for egg production was still to be invested during the nesting season, and yolk from non-dominant follicles would not have provided sufficient energy for most females to complete yolk deposition. These insights into follicular dynamics and nutrient provisioning clarify the ongoing reproductive investments made by female green turtles at Tortuguero.

## Introduction

The pathway through which yolk is produced and stored has been conserved throughout the evolution of oviparous vertebrates (Wahli *et al.,* 1981; Polzonetti-Magni *et al.,* 2004; Wu *et al.,* 2013). During vitellogenesis, yolk precursor vitellogenin (VTG) is secreted by the liver under the influence of oestrogen and deposited into ovarian follicles that envelope the female gametes (oocytes). VTG provides most of the nutrients contained in the yolk that nourishes growing embryos and its sequestration is the main cause for the increase in diameter of ovarian follicles prior to ovulation (Wahli *et al.,* 1981; Wallace, 1985; Polzonetti-Magni *et al.,* 2004; Wu *et al.,* 2013; Price 2016).

**Figure 1:**
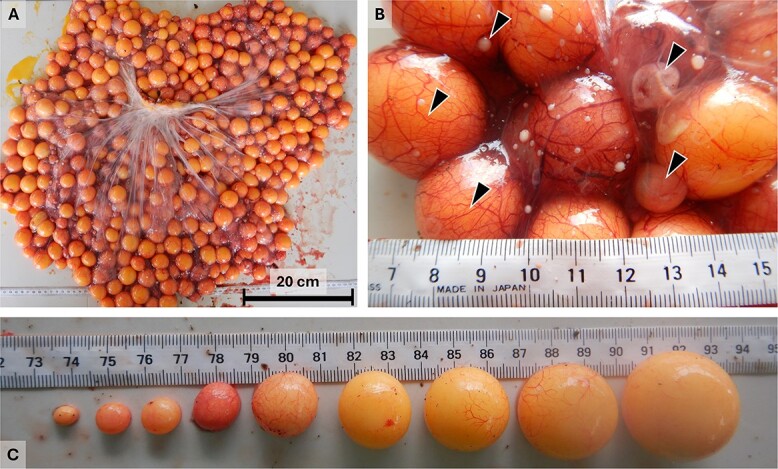
A, One green turtle ovary during the nesting season at Tortuguero. B, Enlarged image of a green turtle ovary. Clockwise from top right, arrows show a recently formed ovulatory scar on top of a fluid-filled vesicle, a non-dominant follicle, a small dominant follicle, a large dominant follicle and a pre-vitellogenic follicle. C, Size hierarchy of ovarian follicles in the ovary of a Tortuguero green turtle. From left to right, the first five follicles are non-dominant follicles that were undergoing atresia. The last four follicles to the right represent the size hierarchy in dominant follicles with the largest follicle at the pre-ovulatory size, to the right.

Sea turtles are oviparous vertebrates that lay multiple clutches of ~100 eggs each nesting season. The reproductive cycle of sea turtles is energetically demanding, encompassing vitellogenesis, reproductive migrations, courtship, mating and nesting. To sustain these costly processes, female sea turtles rely on 2- to 3-year remigration intervals at foraging grounds to replenish the energy reserves necessary for future nesting efforts (Troëng and Chaloupka, 2007).

In sea turtles, vitellogenesis begins 8–12 months prior to the start of the nesting season (Wibbels *et al.,* 1990; Hamann *et al.,* 2002; Miller and Limpus, 2003). Under the influence of follicle-stimulating hormone, hundreds of pre-vitellogenic follicles are recruited throughout the sea turtle ovary and start sequestering VTG (Owens, 1980; Owens and Morris, 1985; Wibbels *et al.,* 1990; Hamann *et al.,* 2002; Miller and Limpus, 2003; Bruno *et al.,* 2021). During this time, vitellogenic follicles will grow from ~3 mm to >30 mm in diameter due to yolk deposition (Miller and Limpus, 2003; Pérez-Bermúdez *et al.,* 2012). The colour of follicles changes from an opaque white of pre-vitellogenic to a warm yellow of vitellogenic follicles (Fig. 1). Ultimately, a vitellogenic follicle will either be ovulated to form an egg or enter follicular atresia—a process in which yolk is resorbed from the follicle so the nutrients invested can be used by the female (Miller and Limpus, 2003; Price 2016).

During ovulation, oocytes are ejected from follicular packages into the oviduct where they are fertilized and covered with layers of albumin and a calcareous shell to form the eggs in a clutch. After ovulation, the ovarian tissue collapses around the ovulated oocytes forming crater-like scars and fluid-filled vesicles (Miller and Limpus, 2003).

Vitellogenesis is believed to be complete prior to reproductive migration in leatherback (*Dermochelys coriacea*) and Kemp’s ridley turtles (*Lepidochelys kempii*) (Rostal *et al.,* 1990, 1996, 1997, 1998; Rostal, 2005). However, a size-based hierarchy of vitellogenic follicles was evident in the ovaries of green (*Chelonia mydas*) and loggerhead turtles (*Caretta caretta*) during the nesting season (Aitken *et al.,* 1976; Limpus and Reed, 1985a, 1985b; Miller *et al.,* 2003). The potential increase in follicular diameter during the nesting season has been posited to be due to the addition of water (Miller *et al.,* 2003). To our knowledge, however, this hypothesis has not yet been tested in any sea turtle species, and high circulating concentrations of VTG indicate that yolk production and/or deposition into sea turtle ovarian follicles may still occur during the nesting season (Blanvillain *et al.,* 2011; Smelker *et al.,* 2014; Bruno *et al.,* 2021).

**Figure 2:**
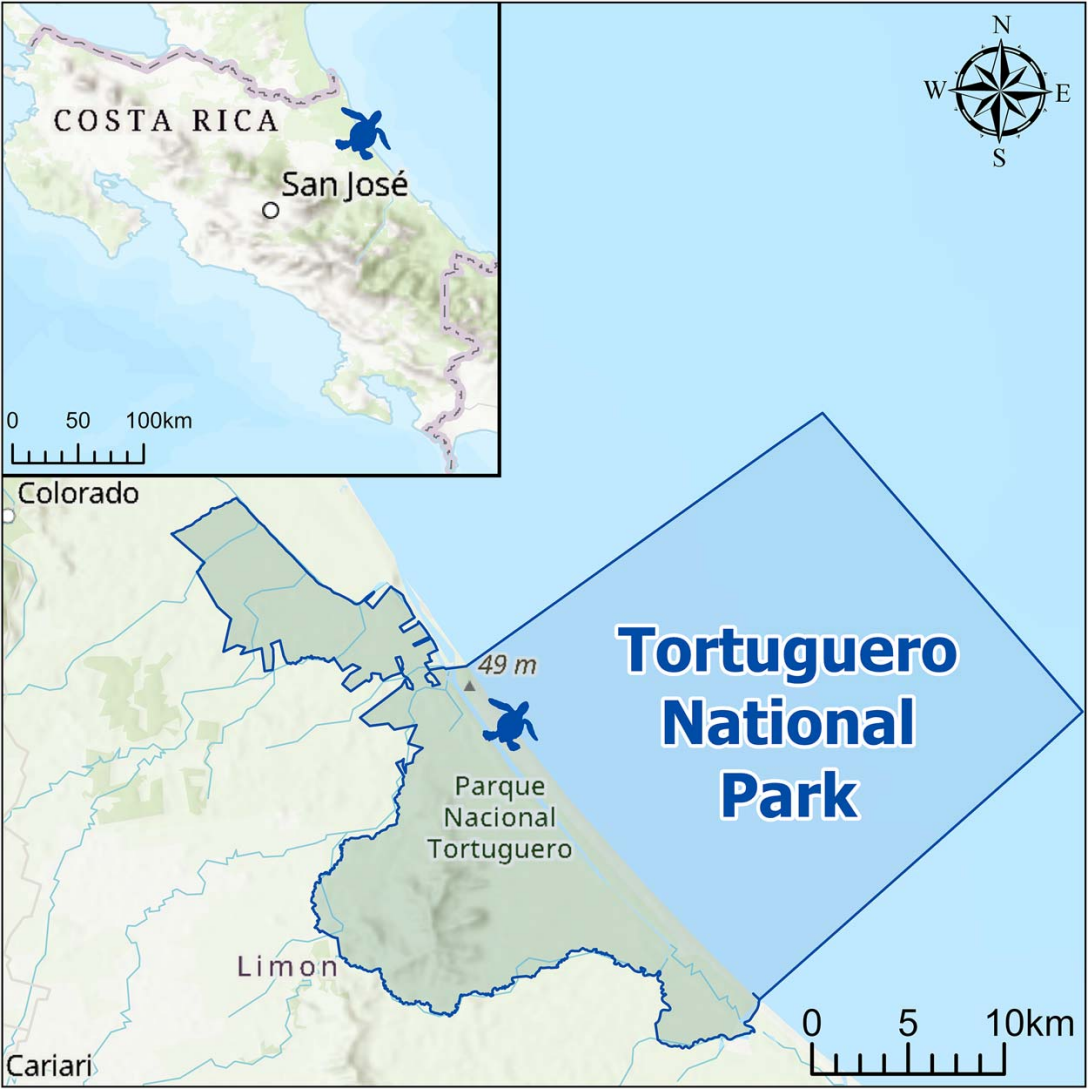
Map shows the location and limits of Tortuguero National Park, which encompasses the main nesting beach for green turtles in the Atlantic Ocean. Map was created in ArcGIS Pro version 3.1.0 (ESRI, 2023) using the base topographic map and the Tortuguero National Park polygon from the World Database on Protected Areas (UNEP-WCMC, 2019). Cartography: R.B.

Given the distinct selective pressures leading to the evolution of sea turtles, the trade-offs shaping female reproductive strategies may vary among species and populations. Therefore, understanding the variation in the parameters of the female green turtle reproductive output, such as the timing of completion of vitellogenesis, will enhance our understanding of the energetic requirements for sea turtle reproduction.

To fill gaps in knowledge, our study addressed three main questions: 1) Are all vitellogenic follicles at maximum size once female green turtles start nesting at Tortuguero? 2) Which nutrients are added to ovarian follicles during the nesting season? 3) Is the energy available for resorption in a green turtle ovary sufficient to complete vitellogenesis for the season? Access to fresh green turtle carcasses allowed us to collect information that was lacking on the basic reproductive biology of sea turtles, such as the size and contribution of each ovary (and ovarian segments) to the reproductive output. Furthermore, we generated a new clutch frequency estimate for this population based on the analysis of ovaries.

## Materials and Methods

### Study site

At Tortuguero, on the Caribbean coast of Costa Rica (Fig. 2), ~100 000 green turtle (*C. mydas*) nests are recorded (Bruno *et al.,* 2020; Restrepo *et al.,* 2023) and >300 nesting green turtles are preyed upon by jaguars (*Panthera onca*) annually (Troëng, 2000; Arroyo-Arce and Salom-Pérez 2015). Jaguars usually attack a sea turtle using bites to the neck; they then pull out the contents of the body cavity with their paws, often leaving ovaries and oviducts undisturbed.

Assisted by National Park rangers and members of the local community, we conducted necropsies of green turtles preyed upon by jaguars at Tortuguero <12 h after the predation event. All activities were conducted under research permits from the Costa Rican Ministry of Environment (SINAC-ACTo-DIR-RES-057-2021, 013–2022, 025–2023).

**Figure 3:**
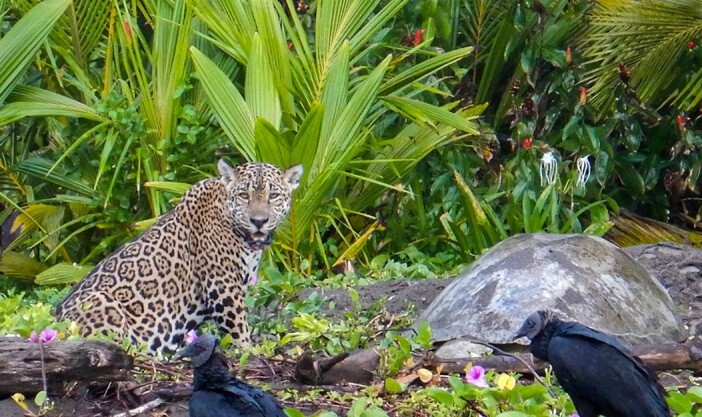
Jaguar (*P. onca*) to the left of a depredated green turtle (*C. mydas*) at Tortuguero National Park, and two black vultures (*C. atratus*) in the foreground. Credits: R.B.

Jaguars eating sea turtles in Tortuguero may return to the kill for several days afterwards to feed (Arroyo-Arce and Salom-Pérez 2015). Therefore, we first identified a predation event from afar by the number of black vultures (*Coragyps atratus*) in the area. Upon finding a depredated green turtle, we monitored the site for 10 min using binoculars to see if the jaguar was still present. If the jaguar was sighted (Fig. 3), we left the area, if not we proceeded to conduct a necropsy. We collected either one or two ovaries from each necropsied turtle and returned to the Tortuguero National Park’s ‘Cuatro Esquinas’ headquarters where we proceeded with analyses.

### Size distribution of ovarian follicles

To determine if all follicles were at maximum size when females arrived at the nesting beach, from 2021 to 2023 we used a calliper to measure the maximum diameter of all vitellogenic follicles (more yellow and larger than pre-vitellogenic follicles). Additionally, we measured all recently formed ovulatory scars, which were evident on top of fluid-filled vesicles (see Fig. 1). We also weighed each ovary to the nearest 500 g and used a graduated cylinder to measure the volume of each ovary by water displacement to the nearest 50 ml.

In 2021, we divided one ovary of each of four females into four segments of similar volume according to the position in the body axis (anterior, mid-anterior, mid-posterior and posterior) and counted follicles and scars separately in each segment (Supplementary Fig. S1). Finally, when possible, we counted the number of eggs in the oviducts of all studied females.

Using R package diptest (Maechler, 2024), we used a Hartigan’s dip test to assess if the distribution of the diameters of green turtle vitellogenic follicles was bimodal. We also used two methods to determine the optimal number of clusters for k-means clustering: the elbow method (Syakur *et al.,* 2018) and the silhouette method (Kodinariya and Makwana, 2013). We then performed k-mean clustering to further analyse the distribution of green turtle ovarian follicles.

Based on results of the k-mean clustering, we classified follicles >18 mm as ‘dominant’. Follicles <18 mm in diameter were classified as ‘non-dominant’ (see Results). We assumed dominant follicles would have been ovulated if the female green turtle had lived, and we assumed non-dominant follicles would have been resorbed by the end of the nesting season (Hodgen 1982; Etches and Pettitte, 1990; Fortune *et al.,* 1991, 2001; Nielsen *et al.,* 2016; Mayor *et al.,* 2023). As follicles may undergo atresia at any stage of development (Price 2017), some dominant follicles would have probably been resorbed by the end of the nesting season.

The number of eggs remaining in a female that would have been laid in that season (eggs left) was calculated by counting the number of dominant follicles, whereas the number of eggs a female had already laid in the season (eggs laid) was calculated by counting the number of recently formed ovulatory scars. These numbers were then divided by 110, which is the average clutch size for green turtles in this population to calculate the number of clutches laid and clutches left (Bjorndal and Carr, 1989; Bruno, 2019). Further, if we had only measured and counted follicles in one ovary, we assumed that both ovaries contained the same number of scars and follicles and multiplied the result by two to obtain the number of clutches laid and clutches left per female. We tested the assumption that ovaries contribute equally to the green turtle reproductive output in one nesting season (see Results).

We classified female green turtles as being in the first or second half of their individual nesting season based on the proportions of clutches laid and clutches left. Females in the first half of their nesting season had fewer clutches laid than clutches left, while those in the second half had an equal or greater number of clutches laid compared to clutches left. Clutch frequency, defined as the number of clutches deposited by a turtle within a nesting season, was estimated by summing the number of clutches laid and left.

### Nutrient analyses

To understand which yolk components were being added to dominant follicles during the green turtle nesting season, in 2022 and 2023 we collected ~10 large and 10 small dominant follicles and a random sample of atretic follicles (AF) per female. We dried collected follicles at 60°C in a food dehydrator until a stable mass was achieved, which prevented sample decay. Finally, we packaged follicles individually in Whirl Pak bags and stored into 1-l Nalgene bottles for transport. These samples were exported from Costa Rica under CITES permits (2021-CR5515/SJ, 2022-CR5969, 2023-CR6500) and imported into the USA under CITES permits (20US72454/9, 21US72454/9, 23US72454/9). We processed samples in the laboratory of the Archie Carr Center for Sea Turtle Research at the University of Florida.

Using a random number generator, we selected five large (LF) and five small (SF) dominant follicles per female for further analyses along with all the dehydrated AF. We ground each of the selected dried follicles to a uniform particle size using a mortar and pestle. We subsampled ground follicles and quantified lipid content by extraction with hexane in an Ankom system according to manufacturer instructions. Another subsample of the same follicles was used to quantify nitrogen and phosphorus content via a modified Kjeldahl method (Bouchard and Bjorndal, 2000). Finally, using the remainder of the sample, we quantified organic and mineral content of the yolk by combusting the dried samples at 600°C for 8 h. Throughout this study, we express water composition as a percentage of wet mass, while all other nutrient compositions are expressed as percentages of dry mass.

### Nutrient investment and resorption

We measured the initial diameter of each vitellogenic follicle in the ovaries we studied, and we measured dry weight only for the follicles we used for nutrient analyses. Therefore, to understand how much yolk and energy was invested in green turtle ovarian follicles, we first used a linear regression (Supplementary Fig. S2) to predict dry weight (g) of each follicle in the studied ovaries:


$$ Dry\ weight\ (g)\!=\!-10.1+0.572\ast initial\ follicle\ diameter\ (mm) $$

The dry weight of each dominant follicle (predicted or measured) was taken as the yolk investment already made by the female. To estimate the additional yolk (in grams) that would be invested in each follicle during the nesting season, we subtracted the dry weight of each dominant follicle from the weight of the largest dominant follicle in the same ovary. The dry weight of all non-dominant follicles was considered as the yolk available for resorption by the female.

We then calculated the total grams of yolk each female had deposited and would have deposited and resorbed throughout the nesting season. If follicles were measured and counted in both ovaries, we summed the yolk amounts from each ovary. If only one ovary was measured, we multiplied the yolk sum by two to estimate the total yolk investment. Finally, using the average values of lipid, nitrogen and phosphorus per gram of dry yolk specific to each female, we determined the total grams of each nutrient that would be deposited into and resorbed from green turtle ovarian follicles during the nesting season at Tortuguero.

### Statistical analyses

We used R programme version 4.4.0 (R Core Team, 2024) for all statistical analyses, and we generated plots with package ggplot2 (Wickham, 2016). We checked if data were parametric by using a Shapiro–Wilks test in base R to assess normal distribution and a Levene’s test or Bartlett’s test in package car (Weisberg, 2019) to assess data homoscedasticity.

We used a Welch’s *t*-test to investigate whether the number of dominant follicles varied significantly according to the stage of the nesting season each female green turtle was in (first or second half). We used a Wilcox test in package rstatix (Kassambara, 2023) to test if the number of non-dominant follicles differed significantly between the two stages of the nesting season.

We investigated whether nutrient content of yolk was different among follicle types (fixed effect) while blocking for the effect of female id (random effect) in a complete block design. Because of the smaller sample sizes of AF, we first ran these models using all follicle types (AF, SF and LF) and then again using only dominant follicles (SF and LF). For these analyses, when the data did not fit the assumptions for parametric statistics, we used a Durbin test in package PMCMRplus (Pohlert, 2023). In case of a significant result (*P* < 0.05) in the analyses among the three groups, we used a DurbinAllPairs test to understand the variation among groups. When the data fit the assumptions for parametric statistics, we used a general linear mixed effects model (LMM) in the lme4 package (Bates *et al.,* 2015). In case of a significant result (*P* < 0.05) in the analyses among the three groups, we used a Tukey HSD test with a Bonferroni correction to understand the variation among groups.

## Results

### Size distribution of ovarian follicles

**Figure 4:**
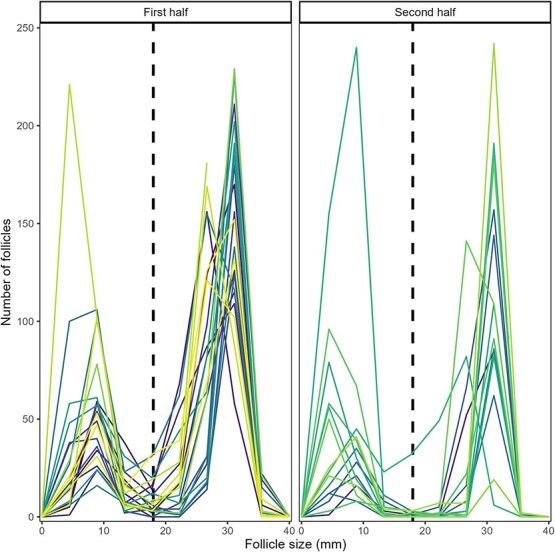
Distribution of ovarian follicles in green turtles across 4-mm size classes at the first (*n* = 20) and second half (*n* = 15) of their nesting season. Each line represents an individual turtle. The data exhibit a significant bimodality, with most AF falling within the diameter range of 5–15 mm and most vitellogenic follicles falling within the range of 20–35 mm. A distinct dip can be observed around 20 mm follicle diameter (D = 0.076; *P* < 0.001).

We counted and measured the diameter of ~15 000 vitellogenic follicles and 10 000 ovulatory scars in the ovaries of 47 green turtles preyed upon by jaguars at Tortuguero between 2021 and 2023. We found a bimodal distribution of diameters of green turtle vitellogenic follicles (Hartigan’s Dip Test D = 0.076; df = 2; *P* < 0.001) (Fig. 4).

Both the elbow and the silhouette methods suggested that two clusters optimally explained the distribution of green turtle vitellogenic follicle diameter (Supplementary Figs S3A and S4A, respectively). Performing k-means clustering with two clusters on all vitellogenic follicles revealed cluster centres at diameters 8 mm (*n* = 4906, x- = 8 mm ± 2.9) and 29 mm (*n* = 9923, x- = 29 mm ± 2.7). Given that the minimum diameter of follicles in the largest cluster was 18.6 mm, we classified all follicles >18 mm in diameter as dominant and assumed these would be ovulated by the end of the nesting season. We assumed that all follicles <18 mm in diameter would have been resorbed by the end of the nesting season.

Using the elbow and silhouette as well as the k-mean clustering methods to understand the size distribution of dominant follicles (Supplementary Figs S3B and S4B, respectively), we identified two clusters centred at diameters 26 mm (*n* = 2892, x- = 26 mm ± 2.1) and 31 mm (*n* = 6648, x- = 31 mm ± 2.9). By calculating the percentage change in diameter for each dominant follicle relative to the largest follicle diameter in the same ovary, we found that the diameter of green turtle dominant follicles increase, on average, 66% during the nesting season at Tortuguero.

There were significantly fewer eggs in the left oviduct (Supplementary Table S1). However, the number of dominant and non-dominant follicles, the number of ovulatory scars and volume did not differ significantly between the right and left ovaries. These results indicate that ovaries may take turns providing more oocytes to each clutch, but both ovaries ultimately contribute a similar number of oocytes during a nesting season. Further, the mean difference between the weight of the left and right ovary was smaller than the accuracy of the scale we used (500 g), so we could not reliably test the significance of the difference (Supplementary Fig. S5). Ovarian volume and weight were positively correlated (Supplementary Fig. S6). The number of follicles and scars per segment of the green turtle ovary were not significantly different (Supplementary Fig. S1B).

**Figure 5:**
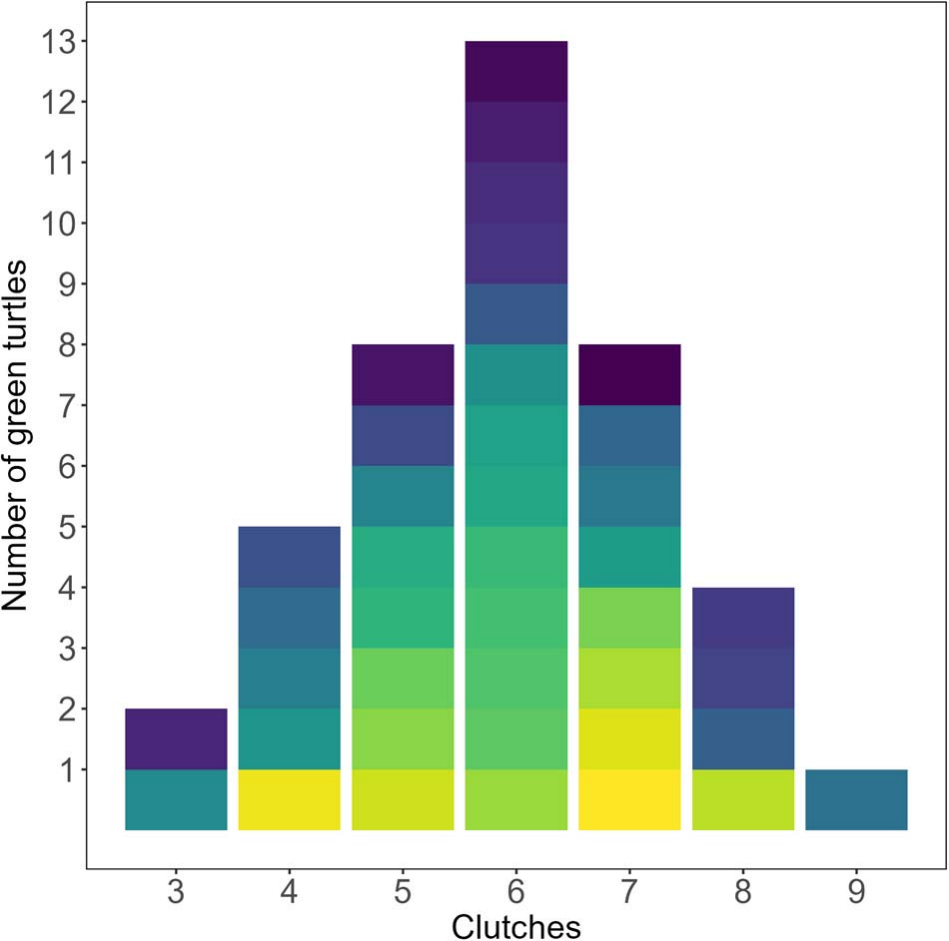
Histogram illustrating the distribution of clutch frequency in green turtles nesting at Tortuguero National Park. Each block in the bars represents an individual female. Clutch frequency varied between 3 and 9 (*n* = 41, x- = 6 ± 1).

**Figure 6:**
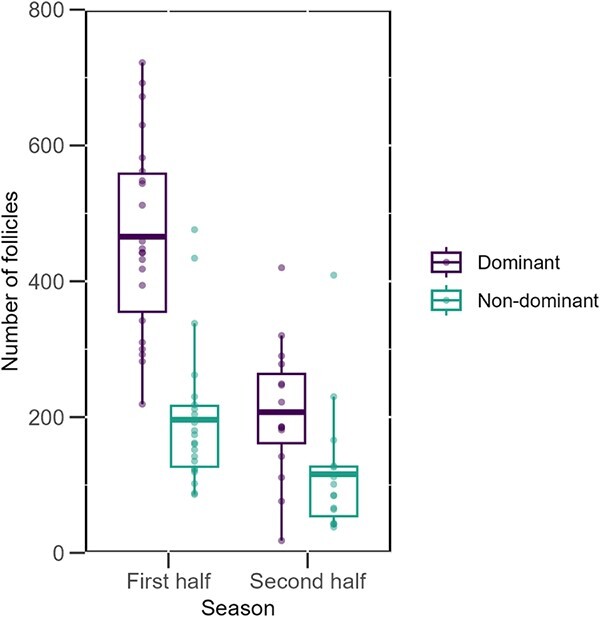
Box plots show the distribution of dominant and non-dominant follicles in the ovaries of green turtles at the first and second half end of their nesting season. Each box plot displays the mean (centreline), interquartile range (box) and whiskers extending to 1.5 times the interquartile range. Individual data points represent the number of dominant and non-dominant follicles in the ovaries of an individual.

Mean clutch frequency of green turtles nesting at Tortuguero between 2021 and 2023 was six clutches per female (ranging from 3 to 9) (Fig. 5). Ovaries of green turtles in the first half of their nesting season had significantly more dominant follicles (>18 mm). Non-dominant follicles (<18 mm) were also significantly more abundant in the ovaries of green turtles in the first half than in the second half of their nesting season (Fig. 6). This indicates dominant follicles were being ovulated and non-dominant follicles were being resorbed throughout the season.

**Figure 7:**
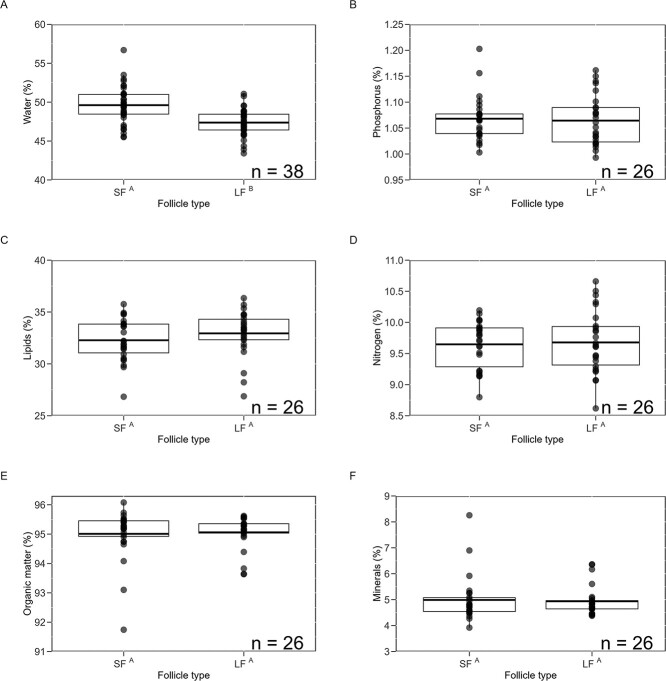
Box plots show the variation in the yolk composition of small (SF) and large (LF) dominant follicles of green turtles nesting at Tortuguero. Water composition is shown as a percentage of total mass, and nutrient composition is shown as a percentage of dry mass. Each box plot displays the mean (centreline), interquartile range (box) and whiskers extending to 1.5 times the interquartile range. Individual data points represent the mean composition of yolk per follicle type per female, with outlying observations plotted beyond the whiskers. Each graph is annotated with the number of females (*n*) for which the analysis was conducted, indicating sample size. X-axis labels that share superscript letters are not statistically different.

### Nutrient analyses

**Figure 8:**
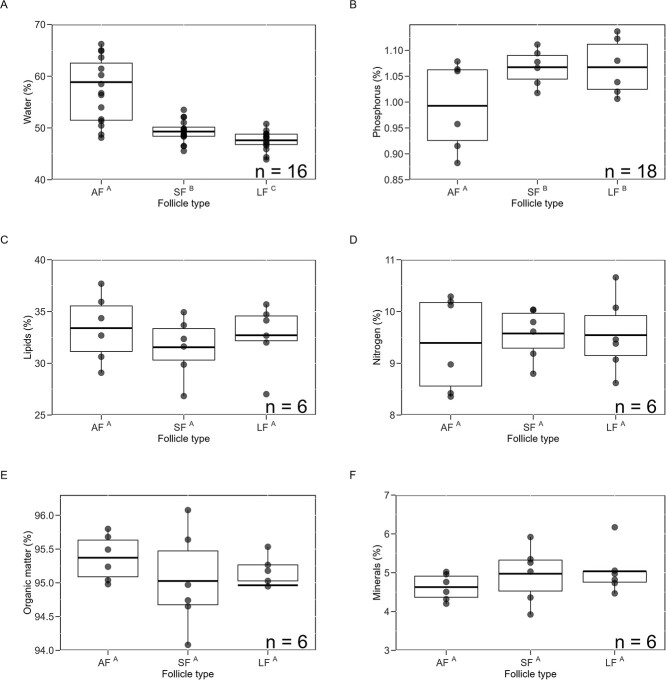
Box plots show the variation in yolk composition among atretic (AF), small (SF) and large (LF) dominant follicles for the green turtles nesting at Tortuguero that represented all three types of follicles. Water composition is shown as a percentage of wet mass, while nutrient composition is shown as a percentage of dry mass. Each box plot displays the mean (centreline), interquartile range (box) and whiskers extending to 1.5 times the interquartile range. Individual data points represent the mean composition of yolk per follicle type per female. Each graph is annotated with the number of females (*n*) for which the analysis was conducted, indicating sample size. X-axis labels that share superscript letters are not statistically different.

There were no significant differences between small and large dominant follicles in percent composition of organic matter, lipids, nitrogen and phosphorus (Fig. 7). There were no significant differences between dominant and AF in percent composition of these nutrients except percent phosphorus content was significantly lower in AF (Fig. 8). AF had higher water percentages compared to both types of dominant follicles. Additionally, small dominant follicles contained significantly higher water percentages than large dominant follicles.

Follicles from 12 females we used for water analyses had to be excluded from the nutrient analyses because samples were damaged during transport. Further, some of the females did not have AF, and sample mass of some follicles was not sufficient for all nutrient analyses. Therefore, number of females and follicles used per analysis varied; sample size and results of each analysis are in Tables 1 and 2.

**Table 1:** Nutrient composition of green turtle ovarian follicles compared by follicle type (AF: atretic follicles, LF: large dominant follicles, SF: small dominant follicles) for females that presented AF. Values presented in bold represent statistically significant differences in nutrient content between follicle types.

Variable	Groups	Females	Follicles	x- (%)	SD	AF-LF	AF-SF	LF-SF
Water	AF	16	43	56.5	8.24	**<0.001**	**<0.05**	**<0.001**
	LF		164	47.8	2.06			
	SF		146	49.6	3.26			
Organic matter	AF	6	19	95.4	0.41	0.168	0.238	0.822
	LF		27	94.9	0.76			
	SF		28	95.0	1.02			
Minerals	AF	6	19	4.62	0.41	0.168	0.238	0.822
	LF		27	5.08	0.76			
	SF		28	5.00	1.02			
Lipids	AF	6	18	32.6	4.95	0.691	0.299	0.508
	LF		26	32.9	3.39			
	SF		28	31.6	2.95			
Nitrogen	AF	6	19	9.47	1.19	0.618	0.547	0.916
	LF		28	9.55	1.10			
	SF		29	9.57	0.95			
Phosphorus	AF	6	18	0.99	0.10	**<0.05**	**<0.05**	0.96
	LF		28	1.07	0.12			
	SF		29	1.07	0.10			

Values are mean percentages ± standard deviation (SD). We used a Durbin Test to assess differences in the water and organic matter composition among follicle types, and we used a linear mixed model to assess differences in lipids, nitrogen, phosphorus and mineral composition. Both statistic tests used nutrient as the dependent variable, follicle type as the independent variable and a block for individual ID. DurbinAllPairs and Tukey HSD *post hoc* tests were used to determine difference between groups, and *P*-values are shown for pairwise comparisons between follicle types (AF-LF, AF-SF, LF-SF).

### Nutrient investment and resorption

**Figure 9:**
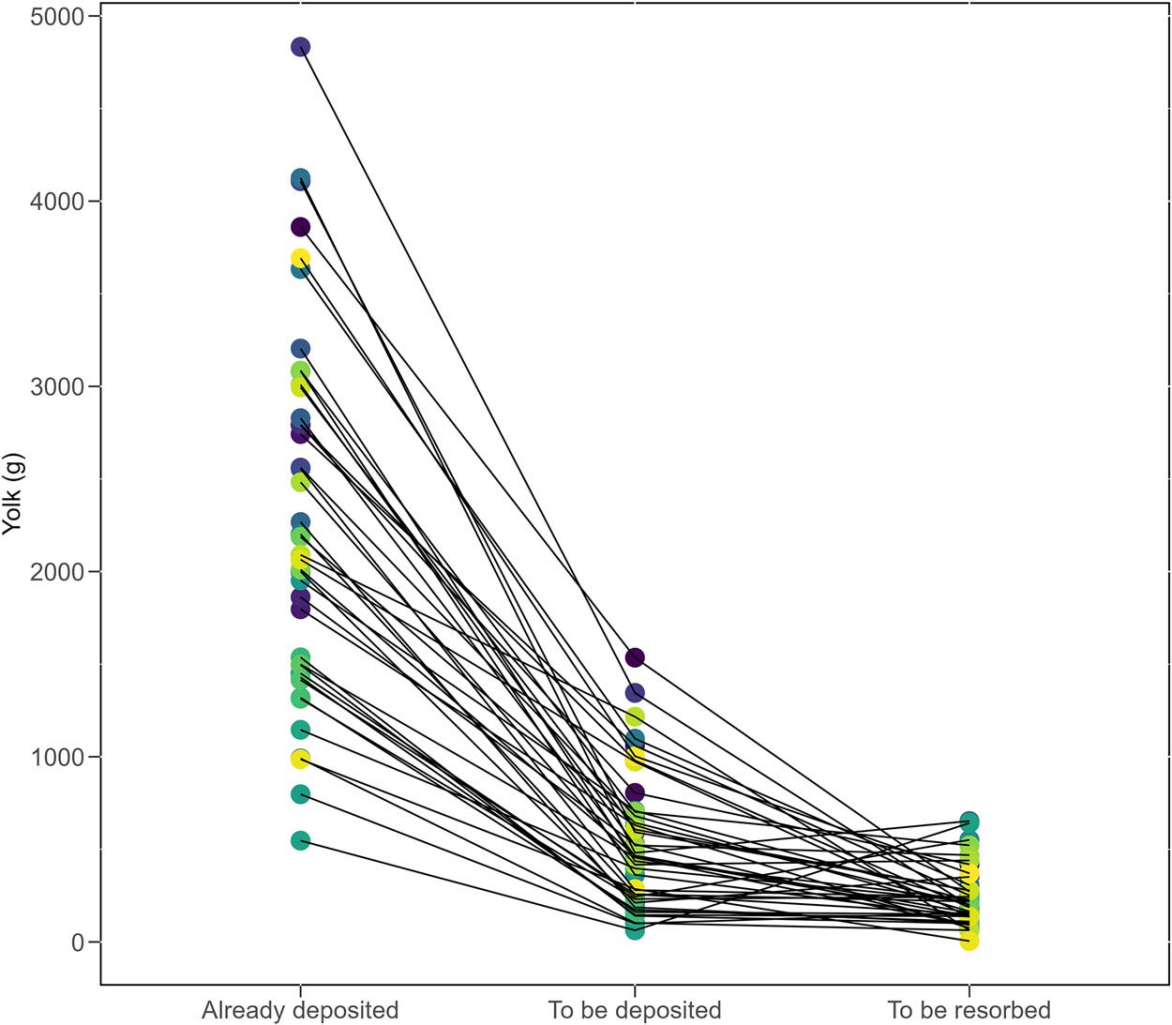
Comparison of total yolk (g) already deposited, to be deposited into dominant follicles, and available to be resorbed from non-dominant. Connecting lines denote measurements from an individual, and coloured points indicate individual IDs.

Based on our female-by-female analysis, we found female green turtles arriving at the nesting beach in Tortuguero had already deposited an average of 2311 g of yolk dry mass into dominant ovarian follicles. Additionally, female green turtles had already invested an average of 239 g of yolk into non-dominant follicles, which would have been resorbed later in the season (Table 3). These turtles were expected to further invest, on average, 523 g of yolk dry mass into dominant follicles during the nesting season. Therefore, 34 out of 41 (83%) female green turtles at the nesting beach needed to deposit more yolk into dominant follicles than what they had available to resorb from non-dominant follicles (Fig. 9).

## Discussion

In this study, we advanced understanding of the reproductive biology of endangered green turtles by examining the dynamics of follicle development and nutrient allocation during the nesting season. Our findings revealed a bimodal distribution of vitellogenic follicles, highlighting a size-based hierarchy within dominant follicles that optimizes reproductive efficiency and space utilization.

Further, the uniform yolk composition across dominant follicles underscores the critical role of nutrient provisioning during the nesting season in supporting embryonic development. Additionally, we identified the energetic demands placed on females, showing that most must mobilize fat stores to sustain their reproductive investment. The role of AF as reservoirs of resorbable yolk provides further insight into the strategies green turtles employ to balance the energetic costs of reproduction. Together, these findings enhance our understanding of how green turtles meet the substantial demands of nesting and offer valuable guidance for conservation strategies aimed at supporting their reproductive health in critical habitats.

### Size distribution of ovarian follicles

We observed that green turtles arriving at the nesting beach in Tortuguero had ovaries containing thousands of vitellogenic and pre-vitellogenic follicles. Pre-vitellogenic follicles, which numbered in the thousands and had an opaque white hue, measured between 1 and 4 mm in diameter. Additionally, vitellogenic follicles, which numbered in the hundreds and had a warmer yellow colour, measured between 4.3 and 37 mm in diameter. Some vitellogenic follicles of all sizes had already started to undergo atresia. This was evident by the penetration of blood vessels into the oocyte (Pérez-Bermúdez *et al.,* 2012) and the colour of the yolk that varied from a reddish to a deep purple tint, depending on the level of atresia (see Fig. 1).

We identified a bimodal distribution of green turtle vitellogenic follicles, with two clusters centred at ~8 and 29 mm in diameter (see Fig. 4). Sea turtle vitellogenesis begins, at the latest, 8 months (240 days) before nesting (Wibbels *et al.,* 1990; Miller and Limpus, 2003), and during this time a vitellogenic follicle may grow from ~3 to 35 mm in diameter. Assuming a constant growth rate, this suggests that follicles increase in size by ~0.13 mm per day. Over a 90-day nesting season, a follicle from the smaller cluster at 8 mm could grow by an additional 12 mm, potentially reaching 20 mm. However, the 21-mm gap between the two clusters (centred at 8 and 29 mm) was too large to be bridged by the addition of yolk to follicles in the smaller cluster during the nesting season.

Because the smallest follicles in the larger cluster measured 18.6 mm, we postulate that follicles >18 mm in diameter would have been ovulated during the current nesting season. Follicles <18 mm in diameter were the green turtle non-dominant follicles and were either being or going to be resorbed (Nielsen *et al.,* 2016; Mayor *et al.,* 2023). Follicular atresia can occur in all stages of follicle development (Makarevich *et al.,* 2011; Price 2017), and some of the green turtle dominant follicles would possibly be resorbed by the end of the nesting season (Miller and Limpus, 2003; Limpus *et al.,* 2005).

**Table 2:** Nutrient composition compared for green turtle dominant ovarian follicles (LF: large dominant follicles, SF: small dominant follicles). Values are mean percentages ± SD. We used a Durbin Test to assess differences in the water and organic matter composition among follicle types, and we used a linear mixed model to assess differences in lipids, nitrogen, phosphorus, organic matter and mineral composition. Both statistic tests used nutrient as the dependent variable, follicle type as the independent variable and a block for individual ID

Variable	Groups	Females	Follicles	x- (%)	SD	*P*-value
Water	LF	38	397	47.4	2.16	**<0.001**
	SF		373	49.7	3.46	
Organic matter	LF	26	124	95.0	1.07	0.695
	SF		125	95.0	1.09	
Minerals	LF	26	124	4.95	1.07	0.635
	SF		125	5.01	1.09	
Lipids	LF	26	121	32.9	3.14	0.077
	SF		125	32.3	2.39	
Nitrogen	LF	26	127	9.68	0.92	0.713
	SF		129	9.65	0.79	
Phosphorus	LF	26	127	1.07	0.10	0.585
	SF		129	1.07	0.09	

**Table 3:** Mean values for yolk deposited, yolk to be deposited and yolk to be resorbed in grams from green turtle ovarian follicles during the nesting season at Tortuguero (*n* = 41). The table presents the mean (x-), SD, minimum (Min), and maximum (Max) values for each metric, which are all reported in grams of dry mass. Nutrient content, including lipids, nitrogen (N) and phosphorus (P), and minerals were calculated by multiplying the mean yolk amount by the respective nutrient content per gram of yolk. Crude protein (CP) was derived by multiplying N by 6.25 (Bouchard and Bjorndal, 2000,), and CP values are also expressed in grams. Energy content was calculated by first converting the maximum yolk amounts to grams of organic matter (multiplying by 0.95) and then converting to kilojoules using a factor of 29.5 (Bjorndal, 1982)

	x-	SD	Min	Max	Lipids	N	P	CP	Minerals	Energy (kJ)
Yolk deposited	2311	1005	547	4833	759.6	224.2	23.7	1400	113	65 000
Yolk to be deposited	523	374	2.3	1535	171.6	50.7	5.2	320	26	15 000
Yolk to be resorbed	239	158	5	653	78.4	23.2	2.4	145	11.8	6700

A size-based hierarchy in the distribution of ovarian follicles is common in vertebrates that produce multiple offspring per clutch/litter or multiple clutches/litters per reproductive cycle (Hamilton and Tam 1990; Nielsen *et al.,* 2016; Song *et al.,* 2023), including turtles (Aitken *et al.,* 1976; Etches and Pettitte, 1990; Mayor *et al.,* 2023). For example, the recruitment of pre-vitellogenic follicles in Guinea pigs (*Cavia porcellus*) happens due to two conspicuous surges of follicle-stimulating hormone a few days apart, which leads to a clear difference in size between follicles recruited during the first and second surges. While follicles recruited first stop growing and are resorbed, follicles recruited later develop fully and are ovulated (Hamilton and Tam 1990). In ball pythons (*Python regius*), Nielsen *et al.* (2016) divided follicles into two groups: follicles that stopped growing halfway through vitellogenesis and were resorbed (non-dominant) and follicles that continued to grow and were ovulated (dominant).

The hormonal triggers for sea turtle vitellogenesis may involve multiple surges of circulating gonadotropins, similar to what has been observed in other taxa such as lizards, where a post-reproductive surge induces the mitotic division of oogonia in the ovaries (Jones, 2011). If green turtles experience a gonadotropin surge during the off-season, it could potentially lead to pre-vitellogenic follicles sequestering the limited VTG available in reproductively quiescent females. This process might also create the size-based hierarchy between dominant and non-dominant vitellogenic follicles. Our findings revealed no significant difference in the number of follicles and scars across different segments of the green turtle ovary, suggesting that recruitment of pre-vitellogenic follicles happens simultaneously across all regions of the ovary. Furthermore, these surges of gonadotropins or the varying sensitivities of individual follicles to hormonal signals that initiate vitellogenesis (Mayor *et al.,* 2023) may also establish the hierarchy we observed among dominant follicles, influencing the sequence in which they are ovulated and, consequently, laid as part of successive clutches.

Our findings demonstrate that the provisioning of dominant follicles with yolk continues after green turtles arrive at the nesting beach in Tortuguero, with smaller dominant follicles increasing in diameter by up to 66% before ovulation. For this green turtle population, the average interval between oviposition events (inter-nesting interval) is ~10 days (Carr *et al.,* 1978; Bjorndal and Carr, 1989; Miller and Limpus, 2003; Troëng *et al.,* 2005), and females typically ovulate again within 24–48 h after having nested (Owens, 1980; Owens and Morris, 1985). Because the turtles we studied were nesting during predation events, ovulation had occurred ~10 days before necropsies were performed. Further, ovulation was expected to occur again within a day or two after the emergence during which the turtle was killed. Consequently, while the diameter of large dominant follicles might have continued to increase, this change was likely minimal due to the brief period between our sample collection and the next anticipated ovulation. In contrast to pre-ovulatory follicles, the size and secretory activity of ovulatory scars decrease with time (Miller and Limpus, 2003), suggesting that the diameter of the newly formed ovulatory scars (formed ~10 days prior to necropsies) was probably larger than what we measured.

One of us (R.B.) had previously examined fresh carcasses of >50 female green turtles from this population legally harvested at their foraging grounds in Nicaragua. When the plastron of these turtles was removed, the gut was immediately visible, with the stomach and intestines filled with food, which obstructed the reproductive organs from view. In contrast, in the fresh carcass of a green turtle nesting at Tortuguero, the predominant features observed were eggs and ovarian follicles, which obscured the digestive tract that was flaccid and contained only liquid and scattered food boli. These observations underscore the nutritional constraint imposed on reproductive females and the potential importance of maternal body size to sea turtle reproductive output as elaborated below.

The strong positive correlation between reptilian maternal body size and clutch/egg size, clutch frequency and offspring mass may be explained by a physical constraint on the female reproductive output (Shine, 2003; Wilkinson and Gibbons, 2005; Mayor *et al.,* 2023). Maternal size is particularly important for the reproductive output of turtles. Gravid females must fit the competing volumes of all organs plus yolked ovarian follicles, a clutch of shelled eggs in the oviducts and perhaps a gut full of food within a coelomic cavity that is limited by a bony carapace (Wilkinson and Gibbons, 2005; Stokes *et al.,* 2019; Mayor *et al.,* 2023).

The size-based hierarchy in the distribution of green turtle ovarian follicles we observed in this study likely optimizes the use of the space available within a female’s coelomic cavity during the nesting season at Tortuguero. Based on our results, ovarian size at the first half of the nesting season would have been up to 63% greater if all follicles were at the size of the largest dominant follicle in that ovary. The largest green turtle ovary we observed during the nesting season in Tortuguero weighed ~8 kg and had a volume of 6 l (16 kg and 12 l if counting both ovaries). If all follicles had been at maximum size from the start of the nesting season, this female would have carried an additional 10 kg or 8 l of volume within its body. While it remains unclear whether the coelomic cavity of a sea turtle could accommodate such an increase in volume, this constraint is likely most relevant at the start of the nesting season when the ovaries and other organs compete for space, and the reproductive system is at its peak demand.

Studies suggesting that yolk deposition in sea turtles is completed prior to the nesting season have primarily focused on Kemp’s ridley and leatherback turtles (Rostal *et al.,* 1990, 1996, 1997, 1998; Rostal, 2005). Kemp’s ridley turtles have an average clutch frequency of fewer than two clutches per season (Shaver *et al.,* 2016), while leatherbacks are significantly larger and possess a more flexible carapace compared to green turtles. These characteristics may allow these species to carry the entire complement of dominant follicles at their maximum size within the coelomic cavity. Further, most of this evidence was based on ultrasound examinations, which provides a limited field of view of the turtle ovary due to the non-echogenic bony carapace and plastron and may not have captured the size-based hierarchy of ovarian follicles.

Because green turtles nesting at Tortuguero will lay multiple clutches (as many as 9), it is surprising that there is not more stratification in the distribution of the dominant follicles, in which each stratum would reflect the follicles to be ovulated for each of the clutches remaining to be laid in that season, as hypothesized by Owens (1980). Similar studies should be conducted in sea turtle species in which the inter-nesting interval is longer, such as the olive ridley (*Lepidochelys olivacea*) (Matos *et al.,* 2012), which may lead to a more pronounced follicular hierarchy than in green turtles. Finally, conducting similar studies in leatherback turtles could further clarify the constraints effected by maternal size and carapace flexibility on the female sea turtle reproductive output.

### Nutrient analyses

If the diameter of follicles increased due to the addition of water only, as posited by Miller *et al.* (2003), large dominant follicles would have had a higher percentage of water than small ones. However, the opposite was true: small dominant follicles had significantly higher percentages of water than large ones. Therefore, the dry components of the yolk rather than water are added to growing dominant follicles during the green turtle nesting season at Tortuguero.

Large and small dominant follicles have similar compositions of organic matter, lipids, nitrogen and phosphorus. These nutrients are added in consistent proportions to growing dominant follicles because the yolk precursor VTG carries the bulk of all nutrients contained in the egg yolk (Wallace, 1985; Polzonetti-Magni *et al.,* 2004).

The higher water and lower phosphorus percentages observed in green turtle AF suggests that phosphorus is prioritized for resorption during follicular atresia, while water resorption is deferred. Sea turtles primarily obtain water through seawater ingestion and excrete excess salts via tear glands and kidneys (Lutz, 1997). In contrast, phosphorus is crucial for eggshell formation and, for females at Tortuguero, would be predominantly sourced from bone hydroxyapatite, which undergoes demineralization to increase phosphorus and calcium levels (Sinclair-Black *et al.,* 2023). The accelerated resorption of phosphorus from AF likely reduces the reliance on bone demineralization for sea turtle eggshell production.

### Nutrient investment and resorption

Bjorndal (1982) reported that the mean egg of a Tortuguero green turtle contained 8.80 g of organic matter, which is equivalent to 259.6 kJ of energy using a conversion factor of 29.5 kJ g^−1^ of organic matter. In our study, we found that large dominant follicles contained an average of 7.73 g of dry yolk, which converts to 7.34 g of organic matter when multiplied by the mean organic matter content of large dominant follicles. Further, using the same conversion factor as Bjorndal (1982), we estimate that an average of 216 kJ is invested in producing each large dominant follicle.

Consequently, while the wet mass of a large dominant follicle represents ~34% of the wet mass of a Tortuguero green turtle egg (Turtle Love *unpublished work*) and 30% of the average wet mass of a green turtle egg reported by Booth and Astill (2001), ~83% of the energy content of an egg is invested in yolk. The remaining 17% are allocated to albumin and eggshell production. This significant investment in yolk is expected, as yolk serves as the primary nutrient source for embryogenesis and the main energy reservoir for hatchling dispersal (Booth *et al.,* 2004).

Based on our data, the female green turtles we studied had already invested an average of 65 000 kJ of energy into dominant follicles (See Table 3), which would have been ovulated. Further, while the average female would have deposited another 15 000 kJ, some females would have invested as much as 43 000 kJ of energy into dominant follicles during the nesting season. The maximum amount of energy invested into yolk to be deposited during the nesting season corresponds to 49% of the total energy investment for egg production and 20% of the overall energy budget allocated for one nesting season (Bjorndal, 1982, 1985). Moreover, while the average female Tortuguero green turtles had deposited 6700 kJ of energy into non-dominant follicles, the maximum amount of yolk available for resorption accounted for ~30 000 kJ.

Another way to understand the energetic investment Tortuguero green turtles make during the nesting season is to consider their fat stores. Female green turtles must acquire, store and mobilize ~5700 g of fat to undergo vitellogenesis, migration, copulation, multiple nesting events at Tortuguero and the subsequent return to foraging grounds while maintaining homeostasis (Bjorndal, 1985). From our study, we estimate that the energy green turtles invest in vitellogenesis during the nesting season accounts for ~1140 g of the total fat mobilized a nesting seasoto fulfill the energetic demands of one full reproductive cycle. Furthermore, energy equivalent to 800 g of fat is available for resorption from non-dominant follicles.

Therefore, our data, analysed on a female-by-female basis, reveal that even if each turtle were to fully resorb all available yolk from non-dominant follicles and invest this energy directly into producing yolk for deposition into dominant follicles, 83% would still experience an energetic deficit by the end of the nesting season. These turtles would need to further mobilize fat stores and/or some of the dominant follicles or acquire additional energy through foraging to meet their energy demands for reproduction (see Fig. 9). Because food ingested at nesting grounds is lower in quality and smaller in quantity compared to the diet of green turtles at the foraging ground (Carr *et al.,* 1974; Mortimer, 1982; Bjorndal, 1985), mobilizing fat stores and resorbing dominant follicles are more likely scenarios than the acquisition of energy through foraging.

There are well-documented mechanisms through which resources invested in producing multiple offspring are redirected to benefit certain individuals, both before and after birth. For instance, in some viviparous sharks, the embryo that develops first in the maternal uterus consumes unfertilized ova or even its own siblings (Miller *et al.,* 2022). Additionally, strategies such as brood reduction, sibling cannibalism and maternal manipulations are mechanisms that favour the survival of some offspring over others (Morandini and Ferrer, 2015). The energy allocation into different size classes of ovarian follicles described in this study can be seen as a pre-emptive version of these phenomena. In this case, the energy invested in the yolk of non-dominant follicles may be reabsorbed and redirected to ensure that female green turtles can successfully complete their nesting seasons.

There is no evidence that yolk resorbed from AF could be deposited directly into other follicles without first being re-synthesized as yolk-precursors. The energy and nutrients resorbed from atretic, and non-dominant follicles may be used for other metabolic costs of the female body during reproduction. Finally, this balance between the yolk to be deposited and yolk to be resorbed could be a cue to end of the nesting season for Tortuguero green turtles.

## Conclusions

In this study we contributed to understanding the reproductive biology of endangered green turtles. By revealing a bimodal distribution of vitellogenic follicles and a size-based hierarchy within dominant follicles, we elucidate how turtles optimize reproductive efficiency and coelomic space utilization. Although most yolk is already invested into vitellogenic follicles when females start nesting, the uniform yolk composition across dominant follicles underscores our observation that nutrient provisioning during the nesting season is essential for embryonic development. Notably, we showed that while the yolk available for resorption in non-dominant follicles is significant, most females must mobilize fat stores to support their reproductive investment. Furthermore, identifying AF as reservoirs of resorbable yolk offers valuable insights into the energetic strategies employed by females during the nesting season.

These findings are critical for informing conservation strategies aimed at supporting the reproductive health and resilience of green turtle populations. Given the tight energetic budgets and substantial demands faced by nesting females, anthropogenic factors that force females to expend unnecessary energy could further disrupt their reproductive success. Addressing these impacts is essential for ensuring the long-term health and stability of green turtle populations at their vital nesting habitats.

## Supplementary Material

Web_Material_coaf012

## Data Availability

The data (https://doi.org/10.5061/dryad.47d7wm3pj) and code (https://doi.org/10.5281/zenodo.13694877) supporting the current study are available in the Dryad Digital Repository and Zenodo.
