# Supplementary material for: Provisioning of vitellogenic follicles continues after green turtles arrive at the nesting beach
Source: Conserv Physiol. 2025 Feb 24;13(1):coaf012. doi: 10.1093/conphys/coaf012 (PMC11858006; doi:10.1093/conphys/coaf012)
Supplement: Web_Material_coaf012 [file web_material_coaf012.pdf]

Provisioning of vitellogenic follicles continues after green turtles arrive at the nesting beach

Renato Bruno,[1,2*] Alan B. Bolten,[1] Karen A. Bjorndal[1]

[1]Archie Carr Center for Sea Turtle Research & Department of Biology, University of Florida, USA

[2] Turtle Love, Barra de Parismina, Limón, Costa Rica

Figures

A

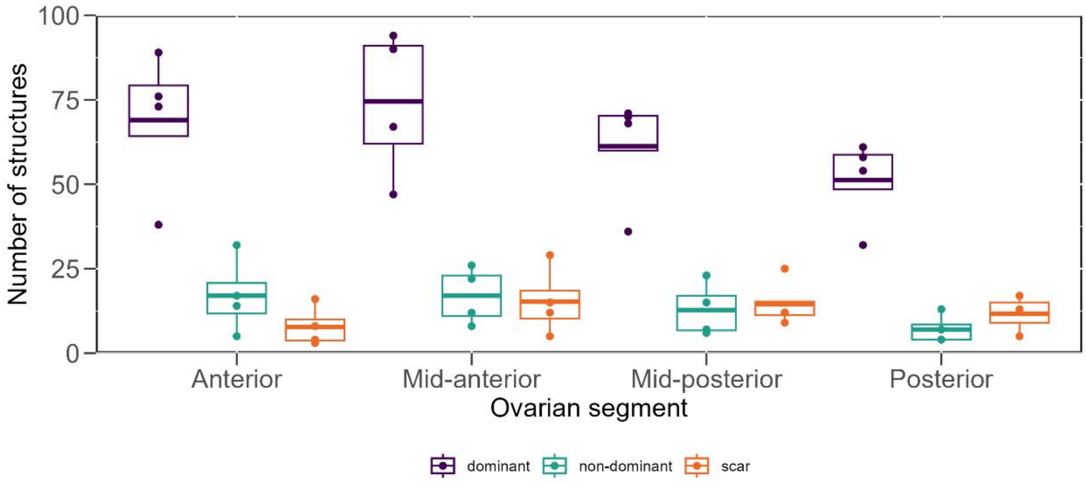

B

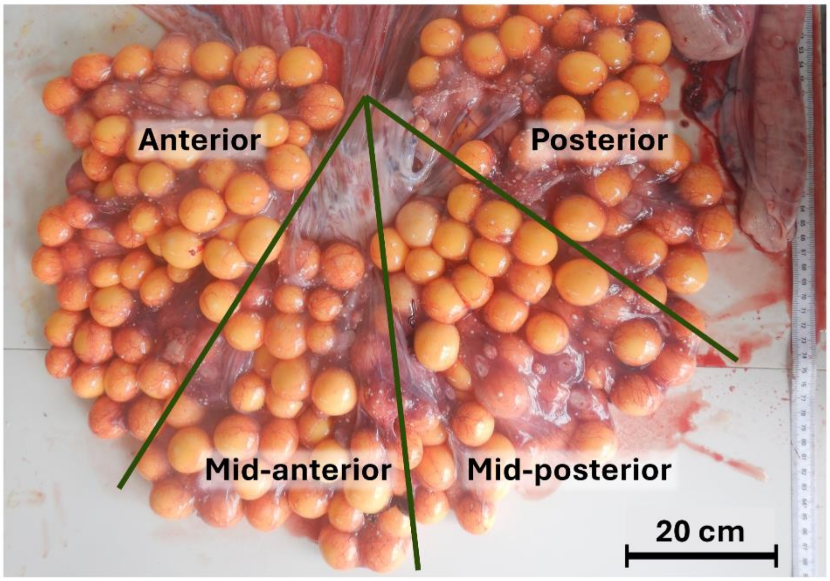

Figure S1. (A) Box plots show the number of structures per ovarian segment in four green turtles during the nesting season. Each box plot displays the mean (center line), interquartile range (box), and whiskers extending to 1.5 times the interquartile range. Individual data points represent individual structure counts. Colors distinguish between dominant follicles, non-dominant follicles, and scars. (B) Image showing a divided green turtle ovary highlighting distinct ovarian segments we used for these analyses.

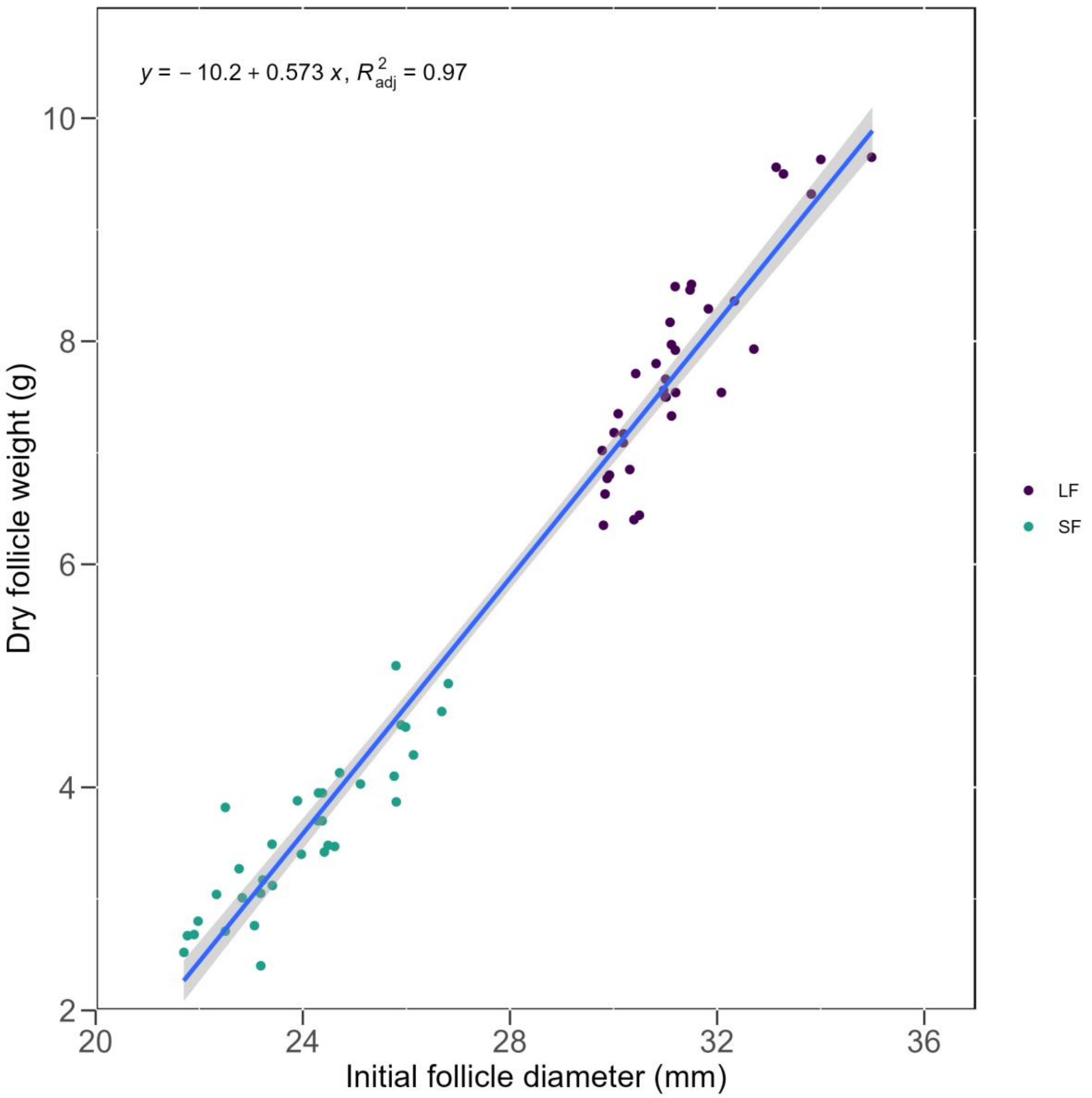

Figure S2. The scatter plot illustrates a strong positive correlation between dry weight and follicle diameter for both small and large vitellogenic follicles ($r^2 = 0.97$, $p < 0.001$). The plot features a regression line with a 95% confidence interval. Points are color-coded to represent the 39 small follicles (SF) and 38 large dominant follicles (LF) analyzed.

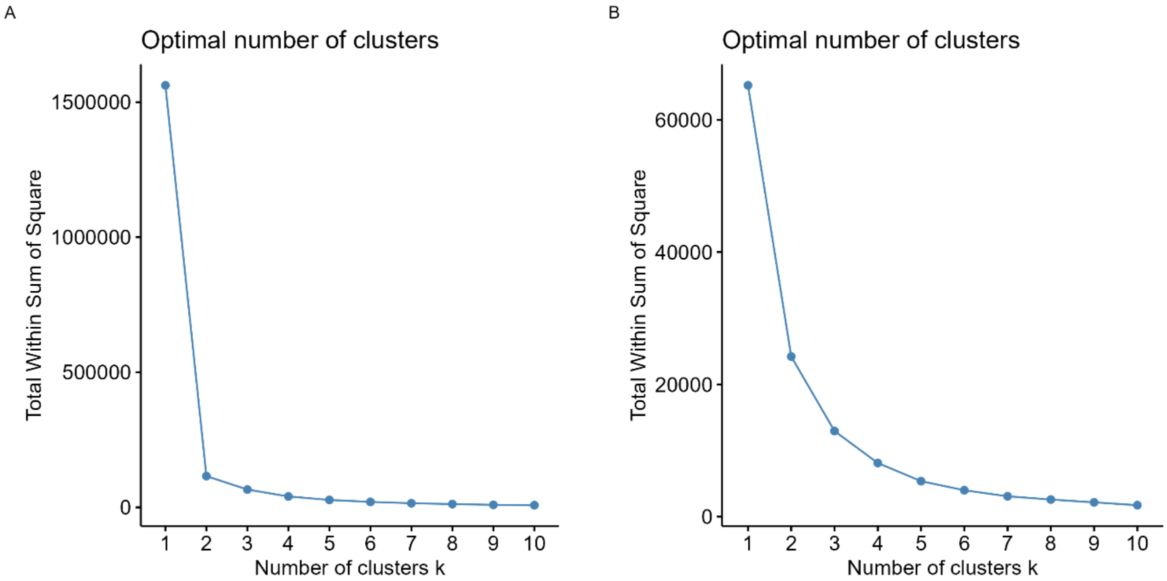

Figure S3. Elbow plot illustrating the within-cluster sum of squares (WSS) values for clustering solutions ranging from 1 to 10 clusters, focusing on the distribution of 14,289 green turtle vitellogenic follicles (A) and of 9,540 green turtle dominant follicles (B) according to their diameter. Each data point represents the total WSS for a specific number of clusters, with the line indicating the trend. The optimal number of clusters, where the decrease in WSS values levels off, indicates the most suitable solution is to cluster the data into two groups.

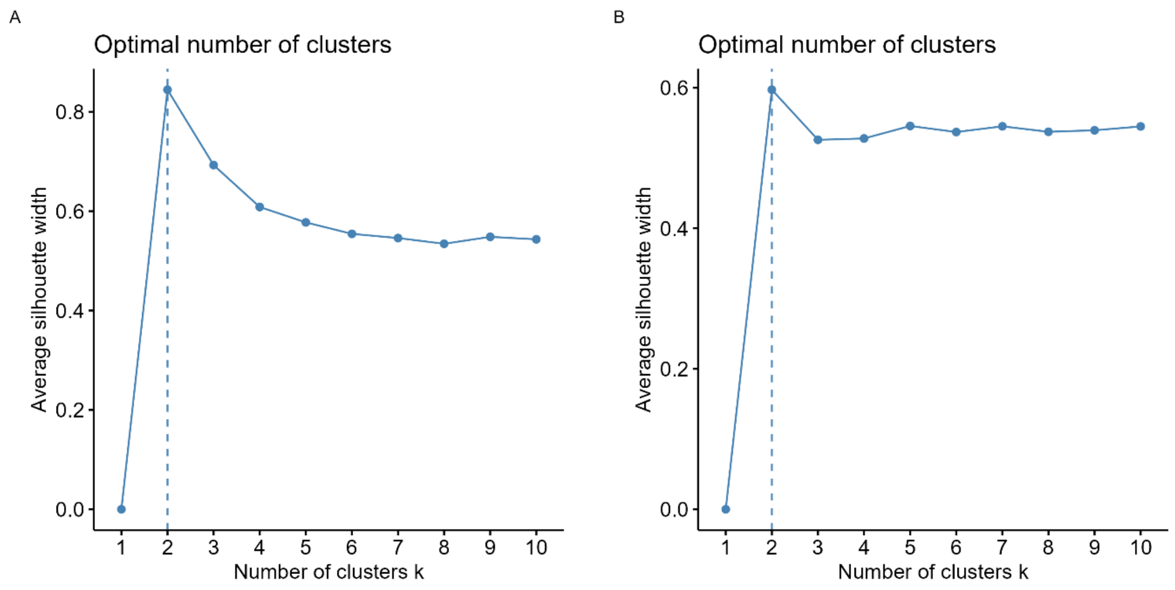

Figure S4. Average silhouette widths for clustering solutions ranging from 2 to 10 clusters, evaluating the diameter distribution 14,289 green turtle vitellogenic follicles (A) and of 9,540 dominant follicles (B). Each point represents the mean silhouette width for a given number of clusters, with a connecting line to illustrate the trend. The optimal number of clusters is indicated by the highest average silhouette width and shown by the dashed line, indicates the most suitable solution is to cluster the data into two groups.

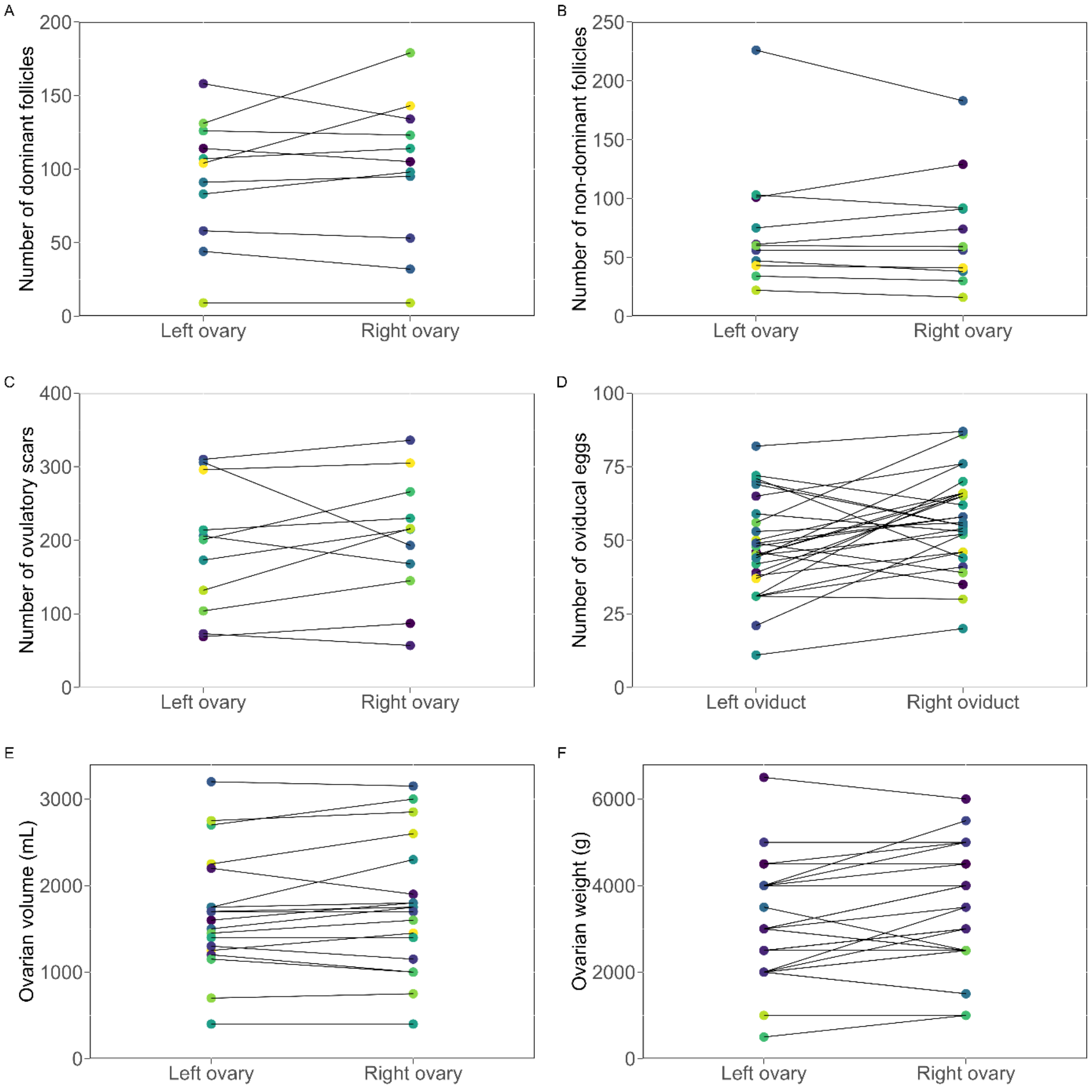

Figure S5. Comparison of reproductive metrics between left and right sides of green turtles. Panels display the number of dominant follicles (A), non-dominant follicles (B), and ovulatory scars (C), showing no significant differences between sides (n = 11). We found significantly fewer eggs ($p = 0.01$) in the left oviduct (n = 29) (D). Ovarian volume (n = 19) (E) shows no significant variation, while ovarian weight (n = 30) (F) indicates lighter left ovaries, although the difference is smaller than the scale's accuracy (500g). Connecting lines denote paired measurements, and colors indicate individual IDs.

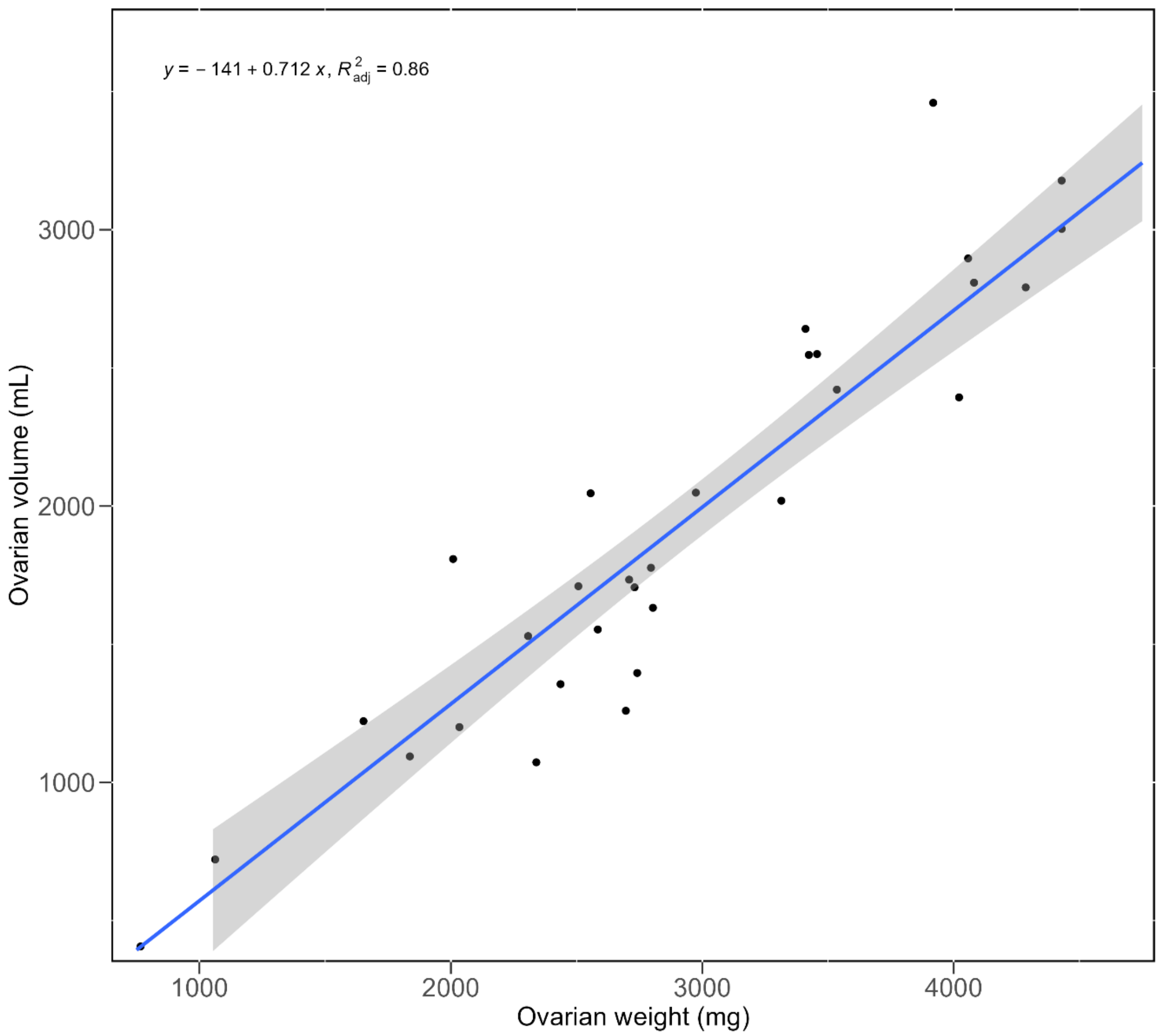

Figure S6. The scatter plot illustrates a significant positive relationship between ovarian volume and ovarian weight in green turtles ($r^2 = 0.86$, $p < 0.001$). The regression line is shown with a 95% confidence interval (n = 47).

Table

Table S1. Results of paired t-tests comparing the number of dominant follicles, non-dominant follicles, ovulatory scars, oviducal eggs, and ovarian volume and weight between the left and right sides of green turtle (*Chelonia mydas*) bodies. N is sample size. Bold p-values are significant.

Variable	N	groups	Mean	SD	df	t-statistic	p
Dominant	11	Left	93	50	10	0.8414	0.4198
follicles	11	Right	99	43			
Non-dominant	11	Left	76	56	10	-0.3161	0.7584
follicles	11	Right	74	49			
Ovulatory	11	Left	189	89	10	0.7516	0.4696
scars	11	Right	202	85			
Oviducal	29	Left	48	16	28	2.7522	**0.0103**
eggs	29	Right	56	16			
Ovarian	19	Left	1682	696	18	1.551	0.1383
Volume (mL)	19	Right	1755	758			
Ovarian	30	Left	3050	1241	29	2.5381	**0.0168**
weight (g)	30	Right	3333	1289			
